# Fish Consumption and Omega-3 Polyunsaturated Fatty Acids in Relation to Depressive Episodes: A Cross-Sectional Analysis

**DOI:** 10.1371/journal.pone.0010530

**Published:** 2010-05-07

**Authors:** Anna Liisa Suominen-Taipale, Timo Partonen, Anu W. Turunen, Satu Männistö, Antti Jula, Pia K. Verkasalo

**Affiliations:** 1 Department of Environmental Health, National Institute for Health and Welfare (THL), Kuopio, Finland; 2 Department of Public Health Dentistry, Institute of Dentistry, University of Turku, Turku, Finland; 3 Department of Mental Health and Substance Abuse Services, National Institute for Health and Welfare (THL), Helsinki, Finland; 4 Department of Chronic Disease Prevention, National Institute for Health and Welfare (THL), Helsinki, Finland; 5 Department of Chronic Disease Prevention, National Institute for Health and Welfare (THL), Turku, Finland; INSERM, France

## Abstract

High fish consumption and omega-3 polyunsaturated fatty acid (PUFA) intake are suggested to benefit mental well-being but the current evidence is conflicting. Our aim was to evaluate whether a higher level of fish consumption, a higher intake of omega-3 PUFAs, and a higher serum concentration of omega-3 PUFAs link to a lower 12-month prevalence of depressive episodes.

We used data from the nationwide Health 2000 Survey (n = 5492) and the Fishermen Study on Finnish professional fishermen and their family members (n = 1265). Data were based on questionnaires, interviews, health examinations, and blood samples. Depressive episodes were assessed with the M-CIDI (the Munich version of the Composite International Diagnostic Interview) and a self-report of two CIDI probe questions, respectively. Fish consumption was measured by a food frequency questionnaire (g/day) and independent frequency questions (times/month). Dietary intake (g/day) and serum concentrations (% from fatty acids) of PUFAs were determined. Fish consumption was associated with prevalence of depressive episodes in men but not in women. The prevalence of depressive episodes decreased from 9% to 5% across the quartiles of fish consumption (g/day) in men of the Health 2000 Survey (p for linear trend = 0.01), and from17% to 3% across the quartiles of fish consumption (times/month) in men of the Fishermen Study (p for linear trend = 0.05). This association was modified by lifestyle; in the Health 2000 Survey a higher level of fish consumption was related to a lower prevalence of depressive episodes in men who consumed the most alcohol, were occasional or former smokers, or had intermediate physical activity. The associations between depressive episodes and the intake or serum concentrations of omega-3 PUFAs were not consistent.

In men, fish consumption appears as a surrogate for underlying but unidentified lifestyle factors that protect against depression.

## Introduction

Depression is currently considered as a complex multifactorial disorder, where the risk factors from multiple domains are related and interacting with each other. In addition to genetic, biological, and environmental risk factors, unhealthy diet - in particular low fish consumption - is suggested to be one risk factor for psychiatric disorders. Low fish consumption and low omega-3 polyunsaturated fatty acid (PUFA) intake are suggested to detrimentally affect various aspects of mental well-being [1], [2]. The effect of fish is thought to be mediated mainly by omega-3 PUFAs that have a role in neurotransmitter synthesis, degradation, release, reuptake and binding [3] but they also suppress inflammation and immune reactivity markers[4]. Research has focused on investigating the role of omega-3 PUFAs, particularly eicosapentaenoic acid (EPA, 20∶5n−3) and docosahexaenoic acid (DHA, 22∶6n−3), in depression, depressive symptoms, and suicidal behaviour.

However, recent reviews conclude that the evidence from clinical trials provide only little support for the claim that omega-3 PUFA supplements reduce depressive symptoms in depressive patients [5], [6], [7], [8]. Epidemiological studies based on non-clinical populations slightly support the inverse relationship between depressive symptoms and fish consumption or omega-3 PUFA intake [2]. However, a recent comprehensive review concluded that for all aspects of mood and behaviour, the current evidence regarding the benefits of high fish consumption or high omega-3 PUFA intake to mental well-being is limited and highly inconsistent also according to epidemiological studies [8]. Inconsistent findings may be due to differences in study designs and methodology, target groups, measures of fish consumption or omega-3 PUFA intake, as well as the selection of confounding factors. Moreover, a variety of measures of depression or depressive symptoms have been used. Clinical studies have investigated individuals with diagnosed clinical depression but the majority of the epidemiological studies have used self-reports of depression or depressive symptoms gathered by diverse questionnaires. At present, little is known about the associations between fish consumption or omega-3 PUFA status and depression in general populations. To our knowledge, there are no previous observational, population-based studies that have utilised M-CIDI interview to study the effects of fish consumption on mental well-being.

Our aim was to evaluate whether higher fish consumption and omega-3 PUFA intake are associated with lower 12-month prevalence of depressive episodes among adults in the Finnish adult population and in a population with high fish consumption.

## Materials and Methods

### Study design

We used two, cross-sectional data sets gathered in Finland. The Health 2000 Survey represented the adult Finnish population aged 30 years and over, and the Nutrition, environment and health study (the Fishermen Study) represented a population with high fish consumption. Ethical approval was obtained from the Hospital District of Helsinki and Uusimaa, and an informed consent from each participant in both studies.

The Health 2000 Survey was carried out in 2000–2001 by the National Institute for Health and Welfare in Finland (THL – which includes the former National Public Health Institute, KTL) [9]. The two-stage stratified cluster sample comprised persons aged 30 years and over (n = 8,028). The sampling frame was regionally stratified according to the five university hospital regions, each comprising roughly one million inhabitants. From each stratum, 16 health centre districts were sampled as clusters: the largest 15 districts in the country were all included and 65 districts were selected by systematic probabilities proportional to size (PPS) sampling. The ultimate sampling units were persons selected by systematic sampling from these 80 health centre districts. The present study population consisted of those participants who attended a structured interview (the Munich version of the Composite International Diagnostic Interview (M-CIDI)) for mental disorders (n = 6,005) [10] during a health examination, had returned a validated, self-administered, semi-quantitative Food Frequency Questionnaire (FFQ, n = 5,998) [11], [12], and had attended an interview including questions about socioeconomics, morbidity, medication, and health behaviour such as smoking and physical exercise (n = 6,986). A sub-sample of persons aged 45–74 years living near five central university hospitals was invited to take further examinations in 2001 and 2002 (Health 2000 Sub-study, n = 1,526). Blood samples were collected and serum levels of fatty acids were analysed. In the initial data checking, participants with mean energy intakes over 6999 kcal or less than 600 kcal (n = 22) where excluded. Additionally, those with missing values for questions on depression (n = 484) were excluded. The effective sample sizes were n = 5,492 in the Health 2000 Survey, and n = 1,265 in the Health 2000 Sub-study

The Fishermen study cohort consisted of all Finnish maritime and freshwater area fishermen, their wives, and other family members. The fishermen were identified from the Professional Fishermen Register [13], whereas the fishermen's wives and other family members (fishermen's biological children, and biological siblings and their spouses and biological children) were identified from the Population Information System of the Population Register Centre. From the registers, we first drew a national random sample of 4,487 fishermen, and their wives and other family members to fill in a questionnaire about fish consumption, morbidity, socioeconomics, medication, and health behaviour such as smoking, physical exercise, and the use of alcohol. From 1,427 respondents, 309 volunteers living near Helsinki and Turku field study locations attended a sub-study conducted between August 2004 and May 2005. Blood samples were collected to measure the serum concentrations of fatty acids. The participants also completed a food-frequency questionnaire (FFQ) similar to that used in the Health 2000 Survey. Subjects with missing values for questions on depression (n = 24) were excluded, the effective sample sizes were n = 1,403 in the Fisherman Study, and n = 308 in the Fishermen Sub-study.

### Measurement of depressive episodes

The study outcome was 12-month prevalence of depressive episodes. In the Health 2000 Survey, a prevalence of Major Depressive Episodes (MDE) was determined by a Finnish translation of the German, computerized version of the M-CIDI interview which was utilised with DSM-IV[10] criteria. The M-CIDI program was translated into Finnish in collaboration with a Munich-based computer operator, who produced Finnish-language M-CIDI versions of different interview components for preliminary testing. Rough translations were tested both at the pilot stage of the survey and at other stages with volunteer subjects. The interview items were translated from English into Finnish by health care professionals with knowledge of DSM diagnostics. These translations were then edited on the basis of the feedback from the pilot stage as well as from lay respondents in test interviews. Once testing with volunteers was completed, the translation was checked for accuracy by an authorized translator. Some further revisions were made to the questions on the basis of this feedback. The final translation and the computer version produced based on that translation were completed shortly before the field stage survey got underway in August 2000 [14]. The quality control interviews were based on the depression component of the M-CIDI mental health interview, the purpose of which is to establish whether the interviewee meets the diagnostic criteria of depression or chronic depression (dysthymia) during the past 12 months. The κ-value obtained for depression was 0.88 (95% confidence interval 0.64–1.00, percentage of agreement 94%) and for dysthymia 0.88 (95% confidence interval 0.64–1.00, percentage of agreement 98%). Based on these results, the inter-interview reliability of the M-CIDI depression components was excellent [15].

Criterion A for the diagnosis of MDE in DSM-IV requires the presence of at least one of two core symptoms (“depressed mood” and “loss of interest”), together with at least three of the other associated “non-core” depressive symptoms. A diagnosis is given if a total of five or more out of nine depressive symptoms are endorsed. Sub-threshold cases are those who have either two to four symptoms or criteria C (“The symptoms cause clinically significant distress or impairment in social, occupational, or other important areas of functioning.”) is not fulfilled, or those who have two to four symptoms and criteria C or E (“The symptoms are not better accounted for by bereavement, i.e., after the loss of a loved one, the symptoms persist for longer than 2 months or are characterized by marked functional impairment, morbid preoccupation with worthlessness, suicidal ideation, psychotic symptoms, or psychomotor retardation.”) are not fulfilled. It has been previously reported that due to dropouts in the Health 2000 Survey, the prevalence of psychiatric disorders was shown to be underestimated to some extent [15]. Therefore we chose MDE with sub-threshold cases to be the primary outcome. Sensitivity analyses were performed using MDE without the sub-threshold cases. In the Fishermen Study, the occurrence of depressive episodes was determined using the CIDI-SF stem questions “During the past 12 months, have you felt sad, blue or depressed for at least two weeks?” and “During the past 12 months, have you lost interest in most things like work or hobbies or things you usually like to do for fun for at least two weeks?” [16]. A person was considered to have depressive episodes if he/she answered ‘yes’ to both questions.

### Fish consumption

The FFQ used both in the Health 2000 Survey and in the Fishermen Sub-study was an updated version of the questionnaire used in the Kuopio Breast Cancer study [12]. It consisted of 128 commonly used or nutritionally important food items and mixed dishes based on the 24-hour dietary recall used in the national FINDIET 1997 Study (n = 2862). Validity of this FFQ has been studied previously, and the results suggested that the data collected using the FFQ meet the requirements of epidemiological studies [11], [12]. The items were grouped under 12 sub-headings one of them being fish dishes. The participants were asked to indicate the frequency of use of each food item. Nine frequency categories ranged from “never or rarely” to “six or more times per day”. The portion sizes were fixed and if possible, specified using natural units (e.g. serving slice, glass, cup). Fish consumption was converted into grams per day by multiplying the food consumption frequency by fixed portion sizes. The ingredients of mixed foods were broken down into their components as well as the contents of different nutrients, including PUFA, alcohol, and energy intake, using the Finnish Food Composition Database *(* http://www.fineli.fi/index.php *)*.

In the Fishermen Study health questionnaire, the respondents were also asked the frequency of use of different fish dishes and fish species [17]. The frequency categories ranged from “not at all” to “almost every day”. From the frequencies, the sums of all fish dishes, fatty fish species, and lean fish species were calculated as times per month. These independent frequency questions were shown to measure fish consumption equally well when compared with the FFQ on whole diet [17].

### Serum concentrations of fatty acid intake

Fasting blood samples were collected to measure serum concentrations of fatty acids in the Health 2000 Sub-study (n = 1,403) and the Fishermen Sub-study (n = 300). Serum total fatty acid composition was analysed using a gas chromatograph (capillary column, flame ionisation detector) [18] and the concentrations were expressed as percentages from the total fatty acid composition. The analyses were performed in the testing laboratory of clinical chemistry at the National Institute for Health and Welfare (THL – which includes the former National Public Health Institute, KTL) in Turku [18].

### Covariates

Information on demographic background (age, sex, education and marital status), self-reported health, medication, and health behaviour was obtained from face-to-face interviews in the Health 2000 Survey and from self-administered questionnaires in the Fishermen Study

Level of education was assessed using information on formal schooling and vocational training, and for the analyses it was divided into three categories: 1) basic, 2) intermediate and 3) higher education. Marital status was divided into five categories: 1) married, 2) cohabiting, 3) divorced or separated, 4) widow and 5) single. Self-reported severe illnesses ever diagnosed by a physician included cancer, myocardial infarction, cerebral stroke, angina pectoris, cardiac insufficiency, diabetes, back pain or illness, bronchial asthma, rheumatoid arthritis, and mental disorder. In the Fishermen Study, the last four previously mentioned illnesses were reported as diagnosed or treated by a physician during the previous 12 months. In the Health 2000 Survey, the participants' Social Insurance Institution cards were during the interview checked to establish to their entitlements to special reimbursement for medicine costs[14]. After this, the respondents were asked to specify the prescription drugs they had used during the last seven days. The responses were checked from packages or prescriptions by the interviewer, and those who reported to have used antidepressants or psycholeptics and psychoanaleptics in combination during the last seven days were categorized as current users of medication for depression or psychiatric disorders. In the Fishermen Study, the participants were asked when did they last use sleeping pills, tranquilizers or medication for depression with answering options 1) during the last week, 2) 1–4 weeks ago, 3) 1–1 2 months ago, 4) over a year ago or 5) never. Those who reported to use any of the asked medication during the last week were considered as current users of medication for depression or psychiatric disorders. Physical activity was assessed using information about physical exercise during leisure time and commuting, and it was categorised as 1) sufficient, 2) intermediate and 3) sedentary. Smoking was categorized into three classes to distinguish between daily, occasional or former, and never-smokers. Those who reported to smoke occasionally or having smoked previously but had quit ≥12 months ago were considered as occasional or former smokers. Alcohol use during the previous 12 months in the Fisherman Study was inquired by a question ‘How often do you have consumed alcohol enough to feel yourself intoxicated?’ and the responses were combined into 1) at least once a week, 2) at least once a month, 3) less frequently or 4) never. In the Health 2000 Survey and the Fishermen Sub-study, weight and height were measured during the health examination to calculate body mass index (BMI; kg/m^2^). In the Fishermen Study questionnaire, self-reported height and weight were used.

### Statistical methods

For the Health 2000 Survey, we used SAS Callable Sudaan software and weights for handling correlated data with unequal sampling probabilities, and for correcting the effects of over-sampling people aged 80 years and over and for non-response. Non-response was accounted for by calibrating the original design weights using post-stratification [14]. The weights were based on post-stratification with sex, age and region. Nutrient intakes were adjusted for total energy intake using the residual method [19].

In both data sets, fish consumption (g/day and times/month) was divided into quartiles separately in men and women. The age-adjusted prevalence of depressive episodes by quartiles of fish consumption was calculated together with 95% confidence intervals (95% CI) by using predicted marginals and logistic regression. Tests of linear trends across increasing quartiles were conducted by a logistic regression model. The statistical significance of the difference in the prevalence of depressive episodes between the data sets and sexes was evaluated with Chi-square tests. Mann-Whitney tests were used to evaluate the statistical significance of the differences in fish consumption (g/day) and fatty acid intake (g/day) between the participants with or without depressive episodes. Ratios of DHA to AA, EPA to AA, and omega-3 to omega-6 PUFAs dietary intake were calculated.

Logistic regression analyses were fitted for the occurrence of depressive episodes and the results are presented in terms of odd ratios (OR), together with 95% CIs. The associations were adjusted for age and energy in the first phase and then further by other variables shown in Table 1. In the Health 2000 Survey, possible effect modification was studied by adding product terms for quartiles of fish consumption with smoking, and physical activity categorised as shown in Table 1, and alcohol consumption (ethanol, g/day) divided into tertiles. The product terms were added one by one into the fully adjusted models. Statistically significant interactions (p<0.01) between fish consumption quartiles and smoking, and physical activity were detected in the Health 2000 Survey women. Due to this, adjusting for smoking and physical activity would have been inappropriate and therefore the fully adjusted odds ratios for 12-month prevalence of depressive episodes are shown only for the men. Stratified analyses by alcohol consumption tertiles, smoking and physical activity were performed in both sexes. In the Fishermen study, models did not converge properly and stratified analyses were omitted. Finally, the consumption of vegetables (g/day), meat (g/day) and fruits (g/day), and the intakes of fat (g/day) and fiber (g/day) were added one by one into the age- and energy-adjusted models in men and women and fully adjusted models in men.

**Table 1:** Distributions of the participants by sex and occurrence of depressive episodes during the previous 12 months 1 in the Health 2000 Survey and the Fishermen Study.

	THE HEALTH 2000 SURVEY				THE FISHERMEN STUDY			
	Men		Women		Men		Women	
	Depressive episodes							
	Yes(n = 186)	no(n = 2,305)	yes(n = 404)	no(n = 2,597)	yes(n = 62)	no(n = 546)	yes(n = 116)	no(n = 679)
Age, years (Mean, SE^2^)	47 (0.9)	51 (0.3)	49 (0.7)	53 (0.3)	43 (1.5)	47 (0.3)	43 (1.3)	46 (0.5)
Level of education (%)								
Basic	29	36	33	38	33	41	22	27
Intermediate	40	39	28	28	49	42	45	35
Higher	31	25	39	34	18	17	33	38
Missing value (n)	(10)		(8)		(6)		(7)	
Marital status (%)								
Married	53	66	57	58	50	61	65	75
Cohabiting	9	12	10	11	13	17	13	12
Divorced or separated	18	7	14	11	13	4	3	3
Widow	4	3	8	11	–	1	5	2
Single	15	12	11	9	24	17	14	8
Missing value (n)	(10)		(7)		(4)		(4)	
Smoking history (%)								
Never smoker	35	37	55	66	36	42	64	65
Occasional or former	34	36	21	18	38	35	18	22
Daily	31	27	24	17	26	23	18	13
Missing value (n)	(10)		(10)		(12)		(11)	
Physical activity (%)								
Sufficient	26	30	35	35	14	30	32	34
Intermediate	32	30	26	30	55	39	38	39
Sedentary	42	40	39	35	31	31	30	27
Missing value (n)	(20)		(48)					
Alcohol (ethanol), g/day (Mean, SE^2^)	10 (1.3)	7.5 (0.3)	3.2 (0.3)	3.0 (0.1)	12 (3.7)^3^	10 (1.1)^3^	3.1 (0.8)^3^	3.8 (0.4)^3^
Missing value (n)	–		(1)		–		–	
Alcohol induced intoxication (%)								
At least once a week	–	–	–	–	10	15	32	38
At least once a month	–	–	–	–	37	44	48	49
Less frequently	–	–	–	–	37	27	16	11
Never	–	–	–	–	16	14	4	2
Missing value (n)					(50)		(93)	
Energy, MJ/day (Mean, SE^2^)	9.8 (0.3)	10 (0.1)	9.0 (0.2)	9.2 (0.1)	8.8 (0.7)^3^	9.9 (0.3)^3^	9.0 (0.4)^3^	8.7 (0.2)^3^
Body Mass Index, kg/m^2^ (Mean, SE^2^)	26 (0.3)	27 (0.1)	27 (0.3)	27 (0.1)	27 (0.2) 4	27 (0.2) 4	26 (0.5) 4	25 (0.2) 4
Missing value (n)	–		(3)		(1)		(18)	
Occurrence of								
Severe illness^5^ (%)	13	17	15	20	14	10	13	11
Missing value (n)	(15)		(13)		(45)		(41)	
Back pain or illness (%)	36	33	31	31	28	20	28	16
Missing value (n)	(11)		(9)		(32)		(38)	
Bronchial asthma (%)	7	7	9	11	2	3	4	4
Missing value (n)	(9)		(8)		(35)		(42)	
Current medication for depression or psychiatric disorders (%)	15	3	17	5	25	7	33	8
Missing value (n)	(281)		(110)		(38)		(60)	

1 In the Health 2000 Survey, major depressive episodes (MDE) measured by a Finnish translation of the German, computerized version of the M-CIDI interview with DSM-IV[10] sub-threshold cases included. In the Fishermen Study, depressive episodes measured by the CIDI-SF stem questions ‘During the previous 12 months, have you felt sad, blue or depressed for at least 2 weeks?’ and ‘During the previous 12 months, have you lost interest in most things like work or hobbies or things you usually like to do for fun for at least two weeks?[16]. A person was considered to have depressive episodes if she/he answered ‘yes’ to both questions.

2 Standard Error.

3 Available only for the Sub-study.

4 Self-report.

5 Cancer, myocardial infarction, cerebral stroke, angina pectoris, cardiac heat insufficiency, diabetes or rheumatoid arthritis. Cancer, myocardial infarction, and cerebral stroke diagnosed by a physician ever in both surveys. Other diseases in the Health 2000 Survey diagnosed by a physician ever, and in the Fishermen Study diagnosed or treated by a physician during the previous 12 months.

## Results

The 12-month prevalence of depressive episodes was somewhat higher in the Fishermen Study than in the Health 2000 Survey (11% vs. 13%, p = 0.040) being lower in the men than in the women (the Health 2000 Survey 8% vs. 14%, p<0.001, The Fishermen Study 10% vs. 15%, p = 0.014). Without the sub-threshold cases (symptoms less than five out of nine or criteria C or E not fulfilled), the prevalence of MDE in the Health 2000 Survey was 5% (in the men 3% and in the women 7%, p<0.001). The characteristics of the participants according to occurrence of depressive episodes separately for the men and the women are shown in Table 1. The demographic characteristics of the participants in both the Sub-studies were similar to the main studies (data not shown).

In the Fishermen Sub-study, men not having depressive episodes reported to consume almost twice as much fish (g/day, p = 0.006) and had higher dietary fatty acid intake (g/day, p = 0.018) compared with those having depressive episodes. In the Health 2000 Survey, there were no differences in fish consumption (g/day) or dietary fatty acid intake (g/day) between those having or those not having MDE either in the men or the women (Table 2).

**Table 2:** Mean (Standard Error) of fish consumption and the intake of polyunsaturated fatty acids (PUFAs) by sex and occurrence of depressive episodes during the previous 12 months^1^ in the Health 2000 Survey and in the Fishermen Study.

	THE HEALTH 2000 SURVEY				THE FISHERMEN STUDY			
	Men		Women		Men		Women	
	Depressive episodes							
	yes	no	yes	no	yes	no	yes	no
Frequency questions on fish consumption (times/month)					**(n = 62)**	**(n = 546)**	**(n = 116)**	**(n = 679)**
Fish	**–**		**–**		11 (1.4)	13 (0.5)	10 (0.8)	9 (0.3)
Fatty fish	**–**		**–**		5.7 (1.1)	5.3 (0.2)	4.9 (0.4)	4.5 (0.2)
Lean fish	**–**		**–**		3.9 (0.7)	5.8 (0.3)	3.7 (0.4)	3.8 (0.2)
FFQ fish consumption (g/day)	**(n = 186)**	**(n = 2,305)**	**(n = 404)**	**(n = 2,597)**	**(n = 12)**	**(n = 130)**	**(n = 29)**	**(n = 136)**
Fish	34 (0.8)	38 (0.8)	36 (1.3)	38 (0.7)	38 (7.9)	70 (5.3)	55 (8.9)	50 (2.3)
FFQ, fish oil supplement user (%)	1	1	3	4	0	5	3	9
FFQ fatty acid intake (g/day)	**(n = 186)**	**(n = 2,305)**	**(n = 404)**	**(n = 2,597)**	**(n = 12)**	**(n = 130)**	**(n = 29)**	**(n = 136)**
Omega-3 PUFAs	2.7 (0.1)	2.7 (0.02)	2.8 (0.04)	2.8 (0.02)	2.8 (0.3)	3.4 (0.1)	3.3 (0.2)	3.2 (0.1)
EPA^2^	0.2 (0.01)	0.2 (0.04)	0.2 (0.01)	0.2 (0.003)	0.2 (0.1)	0.3 (0.02)	0.2 (0.03)	0.2 (0.01)
DHA^3^	0.5 (0.03)	0.5 (0.01)	0.5 (0.02)	0.5 (0.01)	0.5 (0.1)	0.7 (0.1)	0.6 (0.1)	0.6 (0.02)
Alpha-linolenic acid	1.8 (0.01)	1.8 (0.03)	1.9 (0.02)	1.9 (0.01)	1.8 (0.1)	2.1 (0.04)	2.2 (0.1)	2.2 (0.04)
Omega-6 PUFAs	12 (0.2)	12 (0.1)	12 (0.1)	12 (0.1)	9.1 (0.4)	10 (0.2)	11 (0.5)	11 (0.2)
Linolenic acid,	12 (0.2)	11 (0.1)	12 (0.1)	12 (0.1)	7.8 (0.4)	8.8 (0.2)	8.8 (0.4)	9.0 (0.1)
Gammalinolenic acid	0.1 (0.001)	0.1 (0.003)	0.1 (0.001)	0.1 (0.003)	0.1 (0.01)	0.04 (0.002)	0.1 (0.001)	0.1 (0.04)
AA^4^	0.1 (0.003)	0.1 (0.001)	0.1 (0.002)	0.1 (0.001	0.1 (0.01)	0.1 (0.003)	0.1 (0.01)	0.1 (0.002)
Ratio of EPA^2^ to AA^4^	1.6 (0.1)	1.7 (0.03)	1.8 (0.1)	1.9 (0.02)	1.9 (0.4)	2.1 (0.1)	2.3 (0.2)	2.0 (0.1)
Ratio of DHA^2^ to AA^4^	4.1 (0.2)	4.3 (0.1)	4.8 (0.2)	4.9 (0.1)	4.8 (1.0)	5.4 (0.3)	5.8 (0.5)	5.3 (0.2)
Ratio of omega-3 to omega-6 PUFAs	0.2 (0.004)	0.2 (0.001)	0.2 (0.003)	0.2 (0.001)	0.3 (0.02)	0.3 (0.001)	0.3 (0.01)	0.3 (0.01)
Serum fatty acid concentration (% of all fatty acids)	**(n = 45)**	**(n = 525)**	**(n = 92)**	**(n = 603)**	**(n = 12)**	**(n = 127)**	**(n = 28)**	**(n = 133)**
EPA^2^+DHA^3^+DPA^5^	3.8 (0.2)	3.9 (0.1)	3.9 (0.2)	4.0 (0.1)	6.4 (0.9)	7.0 (0.3)	6.6 (0.4)	6.7 (0.2)
EPA^2^	1.1 (0.1)	1.2 (0.03)	1.2 (0.1)	1.2 (0.03)	1.6 (0.3)	2.3 (0.2)	1.6 (0.2)	1.8 (0.1)
DHA^3^	2.1 (0.1)	2.2 (0.04)	2.2 (0.1)	2.2 (0.03)	4.2 (0.7)	4.0 (0.1)	4.4 (0.4)	4.3 (0.2)
DPA^5^	0.5 (0.02)	0.5 (0.02)	0.5 (0.01)	0.5 (0.01)	0.6 (0.04)	0.7 (0.01)	0.6 (0.03)	0.6 (0.02)
AA^4^	4.8 (0.1)	4.4 (0.1)	4.6 (0.1)	4.4 (0.04)	6.1 (0.5)	6.0 (0.1)	6.0 (0.3)	5.9 (0.1)

FFQ = Food Frequency Questionnaire.

1 In the Health 2000 Survey, major depressive episodes (MDE) measured by a Finnish translation of the German, computerized version of the M-CIDI interview with DSM-IV[10] sub-threshold cases included. In the Fishermen Study, depressive episodes measured by the CIDI-SF stem questions ‘During the previous 12 months, have you felt sad, blue or depressed for at least 2 weeks?’ and ‘During the previous 12 months, have you lost interest in most things like work or hobbies or things you usually like to do for fun for at least two weeks?[16]. A person was considered to have depressive episodes if she/he answered ‘yes’ to both questions.

2 Eicosapentaenoic acid.

3 Docosahexaenoic acid.

4 Arachidonic acid.

5 Docosapentaenoic acid.

**Figure 1:**
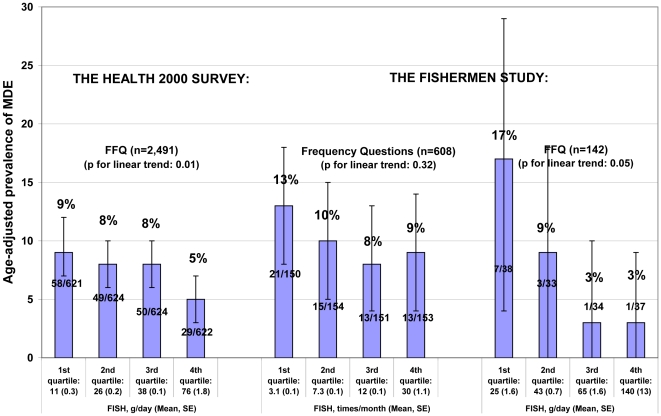
Age-adjusted 12-month-prevalence of depressive episodes in the men by quartiles of fish consumption.

**Figure 2:**
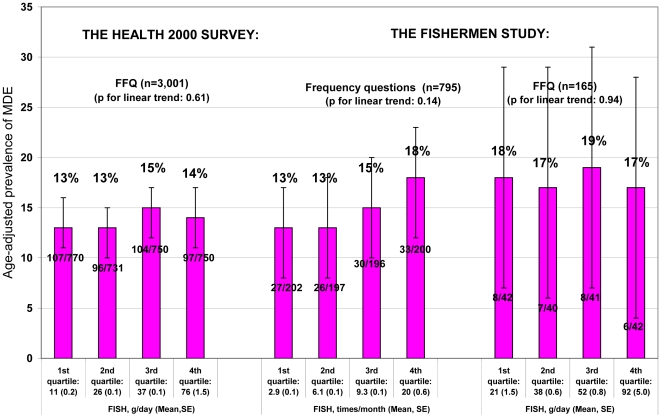
Age-adjusted 12-month-prevalence of depressive episodes in the women by quartiles of fish consumption.

In the men, age-adjusted 12-month prevalence of depressive episodes was the lowest among those who belonged to the lowest fish consumption quartile regardless of the fish consumption measure in both data sets (Figure 1). In the Health 2000 Survey men, the age-adjusted prevalence of MDE without the sub-threshold cases decreased from 4% to 2% across the quartiles of fish consumption (p for linear trend = 0.05). In the women, we observed no consistent associations between fish consumption and age-adjusted prevalence of depressive episodes (Figure 2).

In the men, the adjusted odds ratios for 12-month prevalence of depressive episodes decreased with increasing fish consumption quartiles in both data sets (Table 3). A similar association was seen in the Health 2000 Survey men when using MDE without the sub-threshold cases (the fully adjusted ORs for the highest quartile compared to the lowest one was 0.5 with 95% confidence intervals 0.2–1.2, p for linear trend = 0.090). In the women, we observed no consistent associations. However, in the Health 2000 Survey women, associations were not adjusted for alcohol consumption, smoking or physical activity due to detected interactions. Further adjustment by the consumption of vegetables (g/day), meat (g/day) or fruits (g/day) or by the intake of fat (g/day) or fiber (g/day) did not change the results either in the men or the women.

**Table 3:** Odds ratios with 95% confidence intervals (95%CI) for occurrence of depressive episodes during the previous 12 months^1^ by quartiles of fish consumption (1^st^ quartile as a reference) in the Health 2000 Survey and the Fishermen Study men.

	OR (95%CI)			
	2^nd^ quartile	3rd quartile	4th quartile	p for linear trend
	**THE HEALTH 2000 SURVEY**			
FFQ fish consumption (g/day)	**(n = 2,491)**			
Age and energy adjusted	0.9 (0.6–1.3)	0.9 (0.6–1.3)	**0.5 (0.3–0.9)**	**0.016**
Fully Adjusted^2^	0.9 (0.6–1.4)	0.9 (0.6–1.4)	**0.6 (0.3–1.0)**	**0.030**
Fully Adjusted^3^	0.9 (0.6–1.4)	0.9 (0.6–1.4)	**0.6 (0.3–0.9)**	**0.030**
	**THE FISHERMEN STUDY**			
Frequency questions on fish consumption (times/month)	**(n = 608)**			
Fish				
Age and BMI^4^ adjusted	0.7 (0.4–1.5)	0.6 (0.3–1.3)	0.7 (0.3–1.5)	0.281
Fully Adjusted^2^	0.5 (0.2–1.2)	0.5 (0.2–1.1)	0.5 (0.2–1.1)	0.086
Fully Adjusted^3^	0.6 (0.3–1.3)	0.5 (0.2–1.1)	0.5 (0.2–1.2)	0.109
Fatty fish				
Age and BMI^4^ adjusted	0.7 (0.3–1.5)	0.6 (0.3–1.2)	0.7 (0.4–1.5)	0.316
Fully Adjusted^2^	0.6 (0.3–1.3)	**0.4 (0.2–0.9)**	0.6 (0.3–1.3)	0.119
Fully Adjusted^3^	0.6 (0.3–1.3)	**0.4 (0.2–1.0)**	0.6 (0.3–1.3)	0.144
Lean fish				
Age and BMI^4^ adjusted	1.1 (0.5–2.2)	1.2 (0.6–2.5)	**0.4 (0.2–1.0)**	0.082
Fully Adjusted^2^	1.1 (0.5–2.4)	1.2 (0.6–2.6)	**0.4 (0.2–1.0)**	0.065
Fully Adjusted^3^	1.2 (0.6–2.4)	1.2 (0.6–2.4)	**0.4 (0.1–0.9)**	**0.032**
FFQ fish consumption (g/day)	**(n = 142)**			
Age and energy adjusted	0.5 (0.1–2.1)	0.2(0.02–1.7)	0.1(0.02–1.3)	0.062
Fully Adjusted^2^	0.3 (0.02–3.2)	**0.1(0.01–0.4)**	**0.004(0.0001–0.2)**	**0.002**
Fully Adjusted^3^	0.3 (0.04–1.6)	**0.1(0.01–0.4)**	**0.1(0.02–0.5)**	**0.002**

FFQ = Food Frequency Questionnaire.

1 In the Health 2000 Survey, major depressive episodes (MDE) measured by a Finnish translation of the German, computerized version of the M-CIDI interview with DSM-IV[10] sub-threshold cases included. In the Fishermen Study, depressive episodes measured by the CIDI-SF stem questions ‘During the previous 12 months, have you felt sad, blue or depressed for at least 2 weeks?’ and ‘During the previous 12 months, have you lost interest in most things like work or hobbies or things you usually like to do for fun for at least two weeks?[16]. A person was considered to have depressive episodes if she/he answered ‘yes’ to both questions.

2 Adjusted for age as continuous, total energy intake for fish consumption g/day as continuous, Body Mass Index as continuous (self-reported for consumption of fish times/month), level of education, marital status, smoking history, physical activity, alcohol intake (ethanol, g/day) for fish consumption g/day as continuous, alcohol induced intoxication for consumption of fish times/month, fish oil supplement use for fish consumption g/day, current medication for depression or psychiatric disorders, occurrence of severe illness, bronchial asthma, or back pain or illness.

3 Adjusted as in footnote 2 but without current medication for depression or psychiatric disorders

4 Self-reported Body Mass Index.

Stratified analyses were performed in both sexes by alcohol consumption tertiles, smoking and physical activity. In the stratified analyses, higher fish consumption (g/day) was related to lower prevalence of MDE in the Health 2000 Survey men who consumed the most alcohol, were occasional or former smokers, or had intermediate physical activity. In the Health 2000 Survey women, higher fish consumption was related to higher prevalence of MDE among never-smokers (Table 4).

**Table 4:** Adjusted^1^ odds ratios with 95% confidence intervals (95% CI) for occurrence of major depressive episode during the previous 12 months^2^ by quartiles of fish consumption (1^st^ quartile as a reference) stratified by alcohol consumption tertiles^3^, smoking and physical activity in the Health 2000 Survey.

	MEN				WOMEN			
	Fish, g/day (Food Frequency Questionnaire)							
	2^nd^ quartile	3^rd^ quartile	4^th^ quartile	p for linear trend	2^nd^ quartile	3^rd^ quartile	4^th^ quartile	p for linear trend
Alcohol (ethanol, g/day)^3^								
1^st^ tertile	1.8(0.9–3.7)	1.4 (0.6–3.2)	0.9 (0.3–2.4)	0.739	1.1 (0.6–1.9)	2.4 (1.3–4.3)	1.7 (0.9–3.2)	**0.027**
2^nd^ tertile	0.9 (0.4–2.0)	1.0 (0.5–2.1)	0.9 (0.4–2.0)	0.833	0.9 (0.5–1.5)	0.8 (0.5–1.4)	0.9 (0.5–1.5)	0.556
3^rd^ tertile	**0.5 (0.2–1.0)**	**0.4 (0.2–1.1)**	**0.2 (0.1–0.6)**	**0.006**	1.1 (0.6–1.9)	1.0 (0.6–1.9)	1.4 (0.7–2.6)	0.387
Smoking								
Daily	0.5 (0.2–1.3)	1.0 (0.4–2.5)	1.0 (0.4–2.4)	0.582	0.8 (0.4–1.4)	0.6 (0.3–1.3)	0.7 (0.3–1.5)	0.334
Occasional or former	1.1 (0.5–2.3)	0.7 (0.3–1.7)	**0.4 (0.1–0.9)**	**0.013**	0.6 (0.3–1.9)	0.9 (0.5–1.8)	0.9 (0.4–1.9)	0.863
Never smoker	1.4 (0.6–.3.3)	1.3 (0.6–3.0)	0.7 (0.3–2.1)	0.518	1.5 (0.9–2.4)	**1.9 (1.2–3.0)**	**1.7 (1.1–2.7)**	**0.011**
Physical activity								
Sufficient	1.4 (0.5–3.6)	1.8 (0.8–4.1)	0.8 (0.3–2.4)	0.804	1.2 (0.7–2.2)	1.5 (0.8–2.6)	1.4 (0.7–2.6)	0.266
Intermediate	0.7 (0.3–1.6)	0.8 (0.4–1.5)	**0.3 (0.1–0.8)**	**0.027**	1.1 (0.6–2.3)	1.4 (0.7–2.8)	1.1 (0.5–2.1)	0.691
Sedentary	1.1 (0.6–2.1)	0.7 (0.3–1.5)	1.1 (0.5–2.4)	0.950	0.9 (0.6–1.3)	0.9 (0.5–1.6)	1.0 (0.6–1.7)	0.819

1 Adjusted for age as continuous, total energy intake as continuous, level of education, marital status, smoking history, physical activity, alcohol intake (ethanol, g/day) as continuous, Body Mass Index as continuous, fish oil supplement use, current medication for depression or psychiatric disorders, occurrence of severe illness, bronchial asthma, or back pain or illness.

2 A Finnish translation of the German, computerized version of the M-CIDI interview with DSM-IV[10] sub-threshold cases included.

3 Mean (Standard Error): In men: 1^st^ tertile 0.7 (0.03), 2^nd^ tertile 4.6 (0.1), 3^rd^ tertile 18 (1.0), In women: 1^st^ tertile 0.1 (0.004), 2^nd^ tertile 1.6 (0.02), 3^rd^ tertile 7.4 (0.2).

With regard to omega-3 PUFAs, we observed no consistent associations. In men, the fully adjusted ORs for the 12-month prevalence of depressive episodes were statistically significantly lowered only in the Fishermen Sub-study in the second quartile of serum EPA and in the third quartile of EPA intake (mg/day), DHA intake (mg/day) or omega-3 to omega-6 intake ratio. In the Health 2000 Survey women, the fully adjusted ORs for the MDE were elevated in the third quartile of serum EPA, DHA intake (mg/day), or DHA intake (mg/day), and in the fourth quartile of EPA intake (mg/day).

## Discussion

Our results give some support to the hypothesis that high fish consumption protects against depression. This holds true for the men but not for the women. However, there were no clear associations between omega-3 PUFAs and the occurrence of depressive episodes. Therefore, the beneficial effect in the men may derive from other nutritional compounds than omega-3 PUFAs such as high quality protein, vitamins or minerals. In the men, the association that we observed herein between a higher level of fish consumption and reduced risk for depression indicates a complex association between depression and lifestyle. Fish consumption has been considered as a proxy for a healthier lifestyle that protects against depression. Moreover, we found that high fish consumption suggested to protect against depression those men with high alcohol consumption that is a known risk factor for depression.

In the present study, similar results from the two cross-sectional data sets increase the validity of the results. In addition, both data were comprehensive enough to evaluate the effects of several potential confounding and modifying factors. However, it is not possible to make inferences concerning causality based on these two cross-sectional data sets only. A possibility of causality is supported by a recent review suggesting that it is more likely that low omega-3 PUFAs contribute to a susceptibility to depression rather than depression itself causing changes in either intake or concentrations of lipids [20]. The nationally representative sample of the Health 2000 Survey with high response rate is a clear strength of the present study. Participation rates were high at all stages of the Health 2000 Survey. Almost 85% of the sample attended the health examination which included the M-CIDI-interview and during which the FFQ was delivered. Therefore, selection bias is minimized. Participation in the health examination did not vary by sex and the most active participants among women were those aged 40–69 years and among men those aged 50–79 years. Variation by socioeconomic status was minor but non-participation in the health examination was the highest in the group of respondents who had the least schooling. Mobility restrictions and reduced eye sight also reduced participation especially in the health examination [14]. It is known that people with severe mental health problems participate less often in health surveys leading to underestimation of prevalence of psychiatric disorders which has been shown also in the Health 2000 Survey. Those who attended only the home interview were found to score significantly more symptoms in the Beck Depression Inventory and the General Health Questionnaires Compared to participants in the CIDI interview. They were also older (64 years vs. 52 years), more often single (17% vs. 11%), or widowed (29% vs 9%), and had a low-grade education (65% vs. 39%) [15]. We do not have information regarding non-participants in the Fishermen Study or either of the Sub-studies. In addition, response rate in the Fishermen Study questionnaire was low which reduces the generalisability of the results. However, these data provided an opportunity to use several measures of fish consumption. Additionally, professional fishermen and their families represented a population with high lifelong habitual fish consumption wherein the health effects of fish should most likely be manifested. A limitation of the present study is that both the Health 2000 and the Fishermen Sub-studies were based on convenience samples. Results based on these data cannot be generalized since bias introduced by voluntary participation is possible. In addition, the number of participants was low in the Fishermen Sub-study.

In the Health 2000 Survey, diagnosis of the 12-month prevalence of major depressive episodes (MDE) was based on M-CIDI interview. The CIDI in its several forms has been tested and found acceptable regarding psychometric properties [10], [21], [22]. Moreover, the M-CIDI interview has been tested and found to be valid and reliable tool for assessing psychiatric disorders for large surveys [10], [16], [23]. We used MDE with sub-threshold cases as our primary outcome. However, we repeated the analyses using MDE without the sub-threshold cases and received similar results. In the Fishermen Study, the occurrence of depressive episodes was determined by the two CIDI probe questions, which is not as reliable method as the M-CIDI interview. However, one study has shown that the 2-item measure was as effective as more detailed screening instruments in detecting probable cases for major depression [24].

Current theories suggest a role for omega-3 PUFAs in depression through changes in neuronal membrane structure and function as a results of inadequate omega-3 PUFA dietary intake, abnormal omega-3 PUFA metabolism or increased omega-3 PUFA degradation [25], [26]. We did not find any consistent evidence to support this hypothesis. Although we found some associations between high dietary intake or serum concentrations of omega-3 PUFAs and occurrence of depressive episodes, more often we did not see any associations. Similar lack of association regarding psychological distress was reported earlier in these same data [27]. Methodological issues could be one reason for our results showing no significant associations between omega-3 PUFAs and depressive episodes. Our study used serum concentrations as a biomarker of omega-3 PUFA intake. This particular biomarker is said to mirror the fatty acid consumed over short time periods, ranging from few days to weeks at the very most [28]. It has been reported that fatty acids measured from adipose tissue are better predictors of depression than those measured from serum cholesterol esters in adolescent, adult, and elderly subjects [29], [30], [31], [32] although a recent study failed to replicate this finding when omega-3 PUFAs where assessed from adipose tissue and phospholipids[33]. However, we measured also dietary omega-3 PUFA intake by an FFQ and the result was the same. Thus our findings showing no protective association between both measures of omega-3 PUFAs and depressive episodes are a strong argument against the hypothesis that omega-3 PUFAs are beneficial for mental well-being. This is in line with the previous studies that have evaluated the effect of dietary PUFA intake on depression or depressive symptoms and have found no or non-linear associations [34], [35], [36], [37], [38], [39]. Three studies have shown an inverse association between dietary PUFA intake and depressive symptoms or depressive episodes. In the first study, PUFA intake was evaluated by a dietary history, and outcome of the study was depressive symptoms measured by the Self-rating Depression Scale by Zung [40]. The second study was a cohort study where PUFA intake was evaluated by a dietary record, and antidepressant or lithium prescriptions were used as markers of depressive episodes [41]. The third study using the 20-item Center for Epidemiological Studies Depression Scales and a dietary history including an FFQ found a positive association only in the women [42].

Instead of the omega-3 PUFAs, we found that a higher fish consumption was associated with lower prevalence of depressive episodes in the men but not in the women. A similar association has been found also in two other cross-sectional epidemiological studies which included only men [40], [43] Moreover, in a cohort study on middle-aged French [41], subjects with high fatty fish consumption had a significantly lower risk of any depressive episode and/or recurrent depressive episode, and these associations were stronger in men and in non-smokers as was shown in our study, too. However, the observed association of fish consumption with the risk of recurrent depression was not linear which is again in line with our results. Similar non-linear association without any gender differences was found in another cohort study [35] where moderate consumption of fish was related with lower risk for mental disorder (self-reported physician diagnosis of depression, anxiety or stress, or/and use of antidepressant medication or tranquilizers).

In contrast to our findings, three cross-sectional population based studies together with a one cohort study have shown a positive association between high fish consumption and self-reported mental health or depressive symptoms. One of them found no gender differences [44], and the other three [42], [45], [46] showed significant associations only in women. Two of the studies were population samples from Finland likely with similar food habits and genetic background as in our study. The difference between these two previous Finnish studies and the present study is that the previous ones used frequency questions to measure fish consumption adjusted by BMI. The cohort study investigated self-reported depressive symptoms and the effect was pronounced after 20 year follow-up. In addition, there are two large, Finnish studies on Finnish male smokers aged 50 to 69 years where fish consumption was not found to be associated with anxiety, depressed mood, depression, insomnia, or suicides. Energy-adjusted fish consumption was measured by a dietary history method in the questionnaire based cross-sectional study [36] and by a diet history questionnaire in the cohort study [37] where both self-declared depressive symptoms and hospitalisations for major depression were used as outcomes. These results differ from other Finnish studies, and a possible explanation may be the selected sample of the Alpha-Tocopherol, Beta-Carotene Cancer Prevention study (ABTC study) because of the smoking behaviour of all the studied.

According to our results, the protective effect of high fish consumption against depressive episodes was seen only in the men. This could be due to the fact that the endogenous omega-3 fatty acid concentration is higher in women than in men. One study has shown that with identical diets, plasma DHA concentrations in women are higher than in men [47]. Therefore, high fish consumption increases DHA concentration above some protective level more easily in men. However, we did not see any consistent associations between omega-3 PUFAs and depressive episodes Therefore, fish can contain some other nutritional compounds that have beneficial effects on mental health and these effects are more pronounced in men. These could include high quality proteins or minerals which were not available in our data. In men, high consumption of fish may also be more clearly related to overall healthier diet that may have beneficial effect of mental health. Research on depression has mostly focused on the risk of depression associated with single nutrients. A recent study [48] suggest a protective effect of an overall diet rich in fruits, vegetables, and fish. The potential protective effect of this healthy diet could result from antioxidants and folate in fruits and vegetables. Women probably eat fruits and vegetables more often compared with men even without fish. Association between fish consumption and MDE was modified by health behaviour or lifestyle but modification effect was not consistent. High fish consumption seemed to protect against MDE particularly in men with high alcohol consumption. Alcohol intake has been suggested to be an effect modifier of the relationship between folate intake and plasma homocysteine [49], [50]. Ethanol may affect the absorption and metabolism of folate, leading to a reduction of the potential beneficial effect of folate intake on depression reported in men [51], [52], [53]. Due to the inconsistent findings across the sub-groups, residual confounding cannot be ruled out even though we were able to control the analyses for several confounding factors such as consumption of vegetables and fruits. It is also possible that the lack of protective effect of fish in women is related to reverse causality. Depression may cause changes in overall food consumption in women masking the beneficial effects of fish. Another possible explanation for the gender difference may be that women are more prone to depression than men. The prevalence of depression is about twofold among women compared with men [54], and the beneficial effect of fish might not be strong enough to prevent depression in women.

The observed association between high fish consumption and reduced risk for depressive episodes in the men may indicate complex associations between depression and lifestyle which we were not able to take into account. Diet and fish consumption may be a proxy for factors that have effect on mental well-being particularly in men. A plausible explanation is that fish consumption in men is a surrogate marker for some underlying but yet unidentified lifestyle factors that protect against depression. Therefore, the role of fish consumption as part of the overall diet and lifestyle should be taken into account in future studies.
